# Transfection methods for high-throughput cellular assays of voltage-gated calcium and sodium channels involved in pain

**DOI:** 10.1371/journal.pone.0243645

**Published:** 2021-03-05

**Authors:** Md. Mahadhi Hasan, Lotten Ragnarsson, Fernanda C. Cardoso, Richard J. Lewis

**Affiliations:** Division of Chemistry and Structural Biology, Institute for Molecular Bioscience, The University of Queensland, Brisbane, Qld, Australia; University of Texas Health Science Center, UNITED STATES

## Abstract

Chemical transfection is broadly used to transiently transfect mammalian cells, although often associated with cellular stress and membrane instability, which imposes challenges for most cellular assays, including high-throughput (HT) assays. In the current study, we compared the effectiveness of calcium phosphate, FuGENE and Lipofectamine 3000 to transiently express two key voltage-gated ion channels critical in pain pathways, Ca_V_2.2 and Na_V_1.7. The expression and function of these channels were validated using two HT platforms, the Fluorescence Imaging Plate Reader FLIPR^Tetra^ and the automated patch clamp QPatch 16X. We found that all transfection methods tested demonstrated similar effectiveness when applied to FLIPR^Tetra^ assays. Lipofectamine 3000-mediated transfection produced the largest peak currents for automated patch clamp QPatch assays. However, the FuGENE-mediated transfection was the most effective for QPatch assays as indicated by the superior number of cells displaying GΩ seal formation in whole-cell patch clamp configuration, medium to large peak currents, and higher rates of accomplished assays for both Ca_V_2.2 and Na_V_1.7 channels. Our findings can facilitate the development of HT automated patch clamp assays for the discovery and characterization of novel analgesics and modulators of pain pathways, as well as assisting studies examining the pharmacology of mutated channels.

## Introduction

Chronic pain is a major clinical problem around the globe costing over USD 600 billion per year only in the USA [1]. In order to develop novel and more efficacious analgesics, ion channels participating in pain pathways have been largely investigated. Among these, the voltage-gated ion channels (VGICs), including voltage-gated sodium channels (Na_V_s), voltage-gated calcium channels (Ca_V_s) and voltage-gated potassium channels (K_V_s) are widely distributed in nociceptive nerve fibers and play key roles in pain transmission. In particular, the Na_V_1.1, Na_V_1.3, Na_V_1.6, Na_V_1.7, Na_V_1.8 and Na_V_1.9, Ca_V_2.2, Ca_V_3.2, K_V_1.2, and K_V_1.4 subtypes are expressed in the peripheral and central pain pathways and involved in the initiation and propagation of pain signaling [2–9]. Amongst commercially available pain killers alternative to opioids are drugs that target Ca_V_2.2 and Na_V_ channels, such as Ziconotide and Carbamazepine, respectively, although severe side effects limit their benefits [10, 11]. Hence, the discovery of more effective modulators of these ion channels would benefit the development of novel analgesics to treat chronic pain, as well as assist the understanding of the role of VGICs in pain signaling.

To accelerate the discovery of novel modulators and the pharmacological characterization of VGICs, high-throughput screening (HTS) platforms such as the Fluorescence Imaging Plate Reader (e.g., FLIPR^Tetra^) and automated patch clamp (e.g. QPatch) have been commonly used. Since its introduction in the 1990s, the FLIPR^Tetra^ has been an excellent HTS platform to characterize ion channels and G protein-coupled receptors (GPCRs) through the measurement of changes in intracellular calcium and membrane potential [12–14]. However, electrophysiological methods using cell patch clamping are still considered the gold standard to study VGICs, in spite of lower throughput. To overcome the major hurdle of low throughput conventional patch clamp methods, automated whole-cell patch-clamp instruments such as the QPatch were introduced in the 2000s, which provide high throughput, user friendly and high-quality electrophysiological assays [15]).

A process that plays a pivotal role in expanding research in ion channel HTS drug discovery is the eukaryotic cell transfection. This technique introduces foreign nucleic acids into eukaryotic cells by facilitating their penetration through the cell membrane and translocation to the nucleus, allowing gene function, regulation, and protein expression studies. In stable transfection, plasmid DNA enters to the cell and integrates into the host genome where it is retained after cell division. Unfortunately, stable transfection is generally time-consuming to achieve and hence, more suitable for long-term research and large-scale protein production. In transient transfection, exogenous nucleic acid enters but is not integrated into the host genome and doesn’t survive cell division, and is thus suitable for short-term studies (typically 8–72 h).

**Fig 1:**
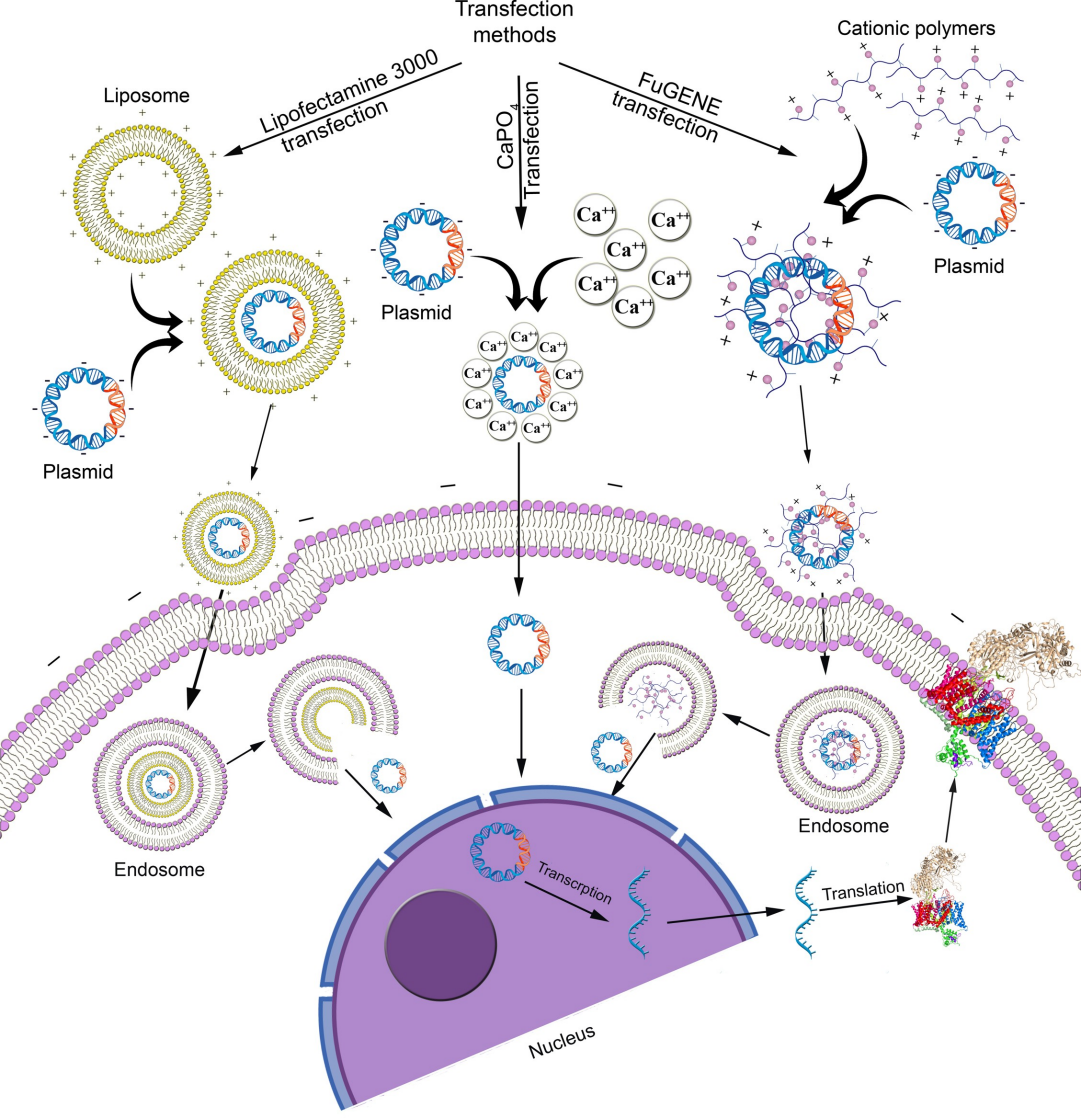
Comparison of chemical transfections.

Transient transfection methods can be classified as chemical, biological, or physical. Chemical transfections use a range of cationic reagents to deliver foreign DNA relatively benignly into cells [16, 17] (Fig 1). In contrast, biological transfection methods introduce foreign nucleic acids into the cells using retrovirus, lentivirus and adenovirus viral vectors [18, 19] with associated immunogenicity and cytotoxicity drawbacks [20]. Recent developed physical transfection methods deliver nucleic acids into cells using microinjection [21], electroporation [22], biolistic particle delivery [23], ultrasound [24] or laser-based transfection [25] but require the skilled use of relatively high-cost instrumentation [20]).

Chemical transfection methods include calcium phosphate (CaPO_4_) precipitation [16, 26], which is inexpensive and readily available, despite its relatively low and variable efficiency [20], liposomal vehicles in which cationic head groups of cationic lipids such as Lipofectamine 3000 attach to anionic phosphates on nucleic acids to form liposomes that cross the cell membrane [27], and non-liposomal transfection reagents such as FuGENE [28] comprising polymers that form complexes with nucleic acid or micelles in aqueous solutions that penetrate the cell membrane. Besides the nature of the transfection reagents, these chemical transfection methods are influenced by factors including nucleic acid/reagent ratio, pH, cell type, confluency of cells, and temperature that can be optimized to enhance the expression of the foreign DNA and maintain cell membrane integrity.

In this current study, we investigated optimum conditions for chemical transfection methods and applied them to express two voltage-gated ion channels, Ca_V_2.2 and Na_V_1.7, into Human Embryonic Kidney 293T (HEK 293T) cells. Furthermore, we used two high throughput platforms, the FLIPR^Tetra^ and the QPatch 16X, to characterize these transiently expressed ion channels and to compare the effectiveness of the calcium phosphate, Lipofectamine 3000 and FuGENE-mediated transfection methods applied to high throughput assays.

## Materials and methods

### Materials

All chemical reagents are from SIGMA Aldrich, USA, unless as stated. The rat Ca_V_ α_1B_ subunit plasmid was a kind gift from Prof David Adams from the School of Medicine, University of Wollongong, AU. The rat Ca_V_ α_2_δ_1_ and rat Ca_V_ β_1_ subunit plasmids were generous gifts from Prof Gerald Zamponi from the Department of Physiology and Pharmacology, University of Calgary, CA. The mouse Na_V_ 1.7 α-subunit plasmid was acquired from GenScript (Hong Kong). The enhanced green fluorescence protein (eGFP) plasmid was a kind gift from Prof Frederic Meunier from the Queensland Brain Institute, The University of Queensland, AU. FuGENE® HD (Promega, USA) and Lipofectamine® 3000 (Thermo Fisher, USA) were used for the transfections. The Ca_V_2.2 inhibitor ω-conotoxin CVID was synthesized in-house by Zoltan Dekan, and the Na_V_1.7 inhibitor tetrodotoxin (TTX) and Na_V_1.7 agonist veratridine were bought from SIGMA Aldrich and Abcam, UK, respectively.

### Cell culture

HEK 293T, CV-1 Origin SV40 (COS-1) and Chinese Hamster Ovary-K1 (CHO-K1) cells were obtained from American Type Culture Collection (ATCC), Virginia, USA. All cell culture reagents are from Gibco, Life Technologies, California, USA, unless as stated. HEK 293T and COS-1 cells were cultured in 5% CO_2_ at 37°C in Dulbecco’s Modified Eagle Medium (DMEM) supplemented with 10% (v/v), and 5% (v/v) fetal bovine serum (FBS), respectively. CHO-K1 cells were cultured in Ham’s F12 Medium (Gibco, Life Technologies) supplemented with 10% (v/v) FBS. Dulbecco’s phosphate-buffered saline (DPBS) was used to wash the cells and 0.25% Trypsin-EDTA used to detach the cells from the flask. The cells were split in a ratio of 1:10 when they reached 70‒80% confluency (every 3‒4 days).

### Transient transfection

For the calcium phosphate-mediated transfection of Ca_V_2.2, 6 μg plasmid DNA of each the α_1B_, α_2_δ_1_ and β_1_ subunit were added in a 1:1:1 ratio to 50 μL CaCl_2_ (2.5 M). dH_2_O was added to make the final volume 500 μL and the mix solution added to 500 μL of 2xHBS buffer (NaCl 274 mM, HEPES 40 mM, Dextrose 12 mM, KCl 10 mM, Na_2_HPO_4_ 1.4 mM). After 15 min incubation at room temperature, the solution was slowly added dropwise to the cells in a T75 flask containing 10 ml fresh medium. For Na_V_1.7 transfection, 10 μg plasmid DNA of the α subunit was added into 30 μL CaCl_2_ (2.5 M). dH_2_O was added to make the final volume 300 μL and the mix solution added to 300 μL of 2xHBS buffer (NaCl 274 mM, HEPES 40 mM, Dextrose 12 mM, KCl 10 mM, Na_2_HPO_4_ 1.4 mM). After 15 min incubation at room temperature, the solution was slowly added dropwise to the cells in a T75 flask containing 10 ml fresh medium.

For the FuGENE-mediated transfection of Ca_V_2.2, 6 μg plasmid DNA of each the α_1B_, α_2_δ_1_ and β_1_ subunit were added in a 1:1:1 ratio to 900 μL serum-free DMEM followed by the addition of 54 μL FuGENE, in a 1:3 ratio of DNA and FuGENE. After 20 min incubation at room temperature, the solution was added dropwise to the cells in a T75 flask containing 10 ml fresh medium. For Na_V_1.7, 10 μg of plasmid DNA encoding the α subunit was added to 300 μL serum-free DMEM followed by the addition of 30 μL FuGENE using a 1:3 ratio of DNA and FuGENE and 20 min incubation at room temperature before the solution was added dropwise to the cells.

**Fig 2:**
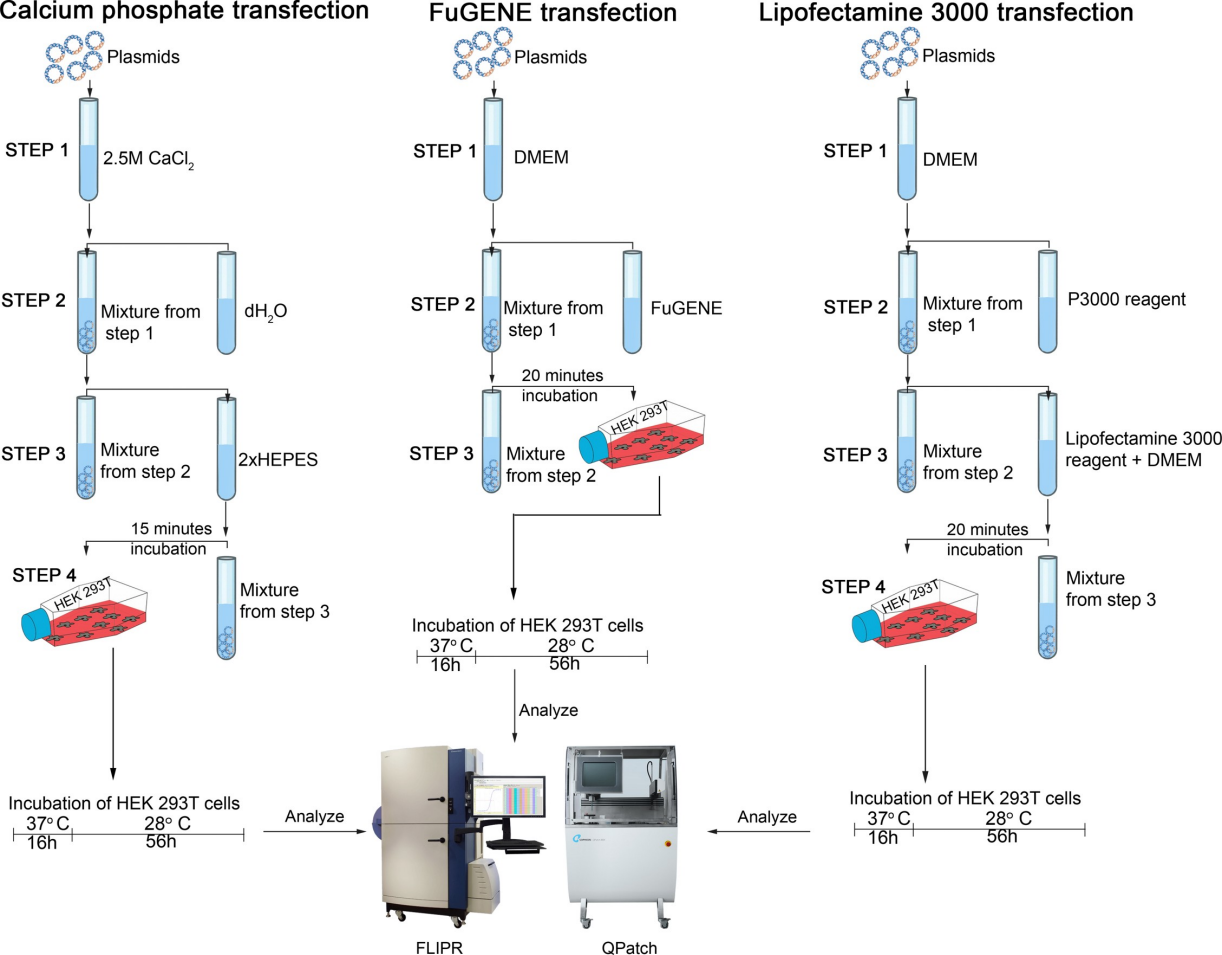
Chemical transfection protocols.

For the Lipofectamine 3000-mediated transfection of Ca_V_2.2, 6 μg plasmid DNA of each the α_1B_, α_2_δ_1_, and β_1_ subunit was added in a 1:1:1 ratio to 500 μL serum-free DMEM followed by the addition of 54 μL P3000 reagent using a 1:3 ratio of DNA and P3000 reagent. This solution was added to another tube containing 27 μL Lipofectamine 3000 reagent mixed to 500 μL serum free DMEM. After 15 min of incubation at room temperature, the solution was dropwise added to the cells in a T75 flask containing 10 ml fresh medium. For Na_V_1.7, 10 μg of plasmid DNA encoding the α subunit was added to 300 μL serum-free DMEM followed by the addition of 30 μL P3000 reagent, in a 1:3 ratio of DNA and P3000 reagent. This solution was added to another tube where 15 μL Lipofectamine 3000 reagent mixed to 300 μL serum-free DMEM. After 15 min incubation at room temperature, the transfection mix was added to the cells. Fig 2 summarizes the steps in each chemical transfection method. As we were interested in comparing ion channel transfection efficiencies, we incubated untransfected cells under the same conditions, instead of transfecting with unrelated cDNA, to obtain a baseline for background calcium response in these cells.

For the eGFP co-transfection, 1 μg eGFP plasmid was added to the Ca_V_2.2 subunits. Transfected cells were visualized using phase-contrast optics on a fluorescence stereomicroscope (M165 FC, Leica, Wetzlar, Germany). Epifluorescence from cells expressing eGFP was excited by the full output of a 75W xenon lamp and observed using the eGFP fluorescence filter set (Leica, Germany), excitation and emission wavelength ranging 470 nm and 525 nm respectively. The transfection efficiency was measured using ImageJ software (National Institute of Health, USA) [29].

### Fluorescence-imaging assays

The increment of intracellular calcium induced by the Ca_V_2.2 channel activation was measured by using calcium-sensitive dye, whereas the changes in membrane potential induced by the Na_V_1.7 activation was measured by using a membrane potential-sensitive dye. Fluorescence signals were detected using a fourth-generation Fluorescence Imaging Plate Reader, FLIPR^Tetra^ (Molecular Devices, Sunnyvale, CA), for both Ca_V_2.2 and Na_V_1.7. 48 hours post-transfection, cells were seeded on 384-well black-walled clear flat-bottom imaging plates (Corning, Lowell, MA, USA) at a density of 30,000 cells/well and maintained for another 24 hours. The dyes (Calcium 4 dye for Ca_V_2.2 and red membrane potential dye for Na_V_1.7, Molecular Devices) were diluted in physiological salt solution (PSS; 140 mM NaCl, 11.5 mM glucose, 5.9 mM KCl, 1.4 mM MgCl_2_, 1.2 mM NaH_2_PO_4_, 5 mM NaHCO_3_, 1.8 mM CaCl_2_, 10 mM HEPES, pH 7.4) with 0.1% Bovine Serum Albumin (BSA). The medium was removed, and the cells were loaded with 20 μL dye per well and incubated for 30 min at 37°C in a 5% humidified CO_2_ incubator.

For the Ca_V_ 2.2 assay, the excitation and emission wavelengths of the FLIPR^TETRA^ were set at 470–495 nm and 515–575 nm, respectively. Camera gain and intensity were adjusted for each plate to yield a minimum of 1500–2000 arbitrary fluorescence units (AFU) baseline fluorescence. A two-addition protocol was used where 5 baseline fluorescence readings were taken prior to the first addition of 10 μL PSS followed by fluorescence readings for 10 min. In the second addition, 10 μL stimulus buffer containing KCl (1–180 mM) and 5 mM CaCl_2_ was added and followed by measurement of fluorescence for another 5 min. To inhibit the agonist activity, CVID (1–500 nM) was used in a two-addition protocol where 10 μL CVID (1–500 nM) was added in the first addition and 10 μL stimulus buffer containing 90 mM KCl and 5 mM CaCl_2_ was added in the second addition.

For the Na_V_1.7 assay, the excitation and emission wavelengths were set at 510–545 nm and 565–625 nm, respectively. For activation, a two-addition protocol was used where 5 baseline fluorescence readings were taken prior to the first addition of 10 μL PSS followed by fluorescence readings for 10 min. In the second addition, 10 μL stimulus buffer containing veratridine (10–60 μM) was added and followed by measurement of fluorescence for another 10 min. To inhibit the agonist activity, TTX (1–1000 nM) was used in a two-addition protocol where 10 μL TTX (1–1000 nM) was added in the first addition and 10 μL stimulus buffer containing 40 μM veratridine was added in the second addition.

### Z’ factor determination of assay robustness

The screening window coefficient, the Z’ factor, was measured to quantitatively represent the FLIPR assay quality by following a previously described method [30]. The Z’ factor was measured from 9 replicates of the negative control (PSS buffer with 0.1% BSA) and 9 replicates of positive controls (90 mM KCl with 5 mM CaCl_2_, or 40 μM veratridine) per plate from three independent assays according to the following equation:

$$Z^{'}=1-\left(\frac{{3\times Standard\;deviation}_{positive}+{3\times Standard\;deviation}_{negative}}{{Mean}_{positive}-{Mean}_{negative}}\right)$$

### Automated whole-cell patch clamp assay

The whole-cell patch-clamp experiments were performed using the automated electrophysiology platform QPatch 16X (Sophion Bioscience A/S, Ballerup, Denmark) in single-hole configuration and after 72 hours post-transfection. The medium was removed, and the cells were washed with DPBS followed by the addition of 2 mL Versene (Thermo Fisher, USA) to detach the cells from the flasks. After a gentle swirl, 1 mL Versene was aspirated out, and cells were incubated in a 37⁰C incubator for ~2 min. The cells were resuspended in 5 mL serum-free medium (EX-CELL ACF CHO medium, 25 mM HEPES, 100 U/mL Penicillin/Streptomycin) to prepare the cell suspension. After cell counting, 6 mL cell suspension containing at least 2 million/mL cells was added to the magnetic stirrer of QPatch (QStirrer) and incubated for 30 min at room temperature before running the assay.

The extracellular recording solution of the Ca_V_2.2 assay contained (in mM): 5.4, 0.4 KH_2_PO_4_, 4.2 NaHCO_3_, 138 NaCl, 0.34 Na_2_HPO_4_, 0.42 BaCl_2_ and 10 HEPES; pH 7.4 adjusted with NaOH; osmolarity 320 mOsm. The intracellular solution contained (in mM): 140 CsCl, 10 EGTA, 10 HEPES, 10 MgCl_2_, and 4 ATP; ATP was added on the day of the experiment; pH 7.2 adjusted with CsOH; osmolarity 325 mOsm. The Ca_V_2.2 inhibitor CVID was diluted in extracellular recording solution containing 0.1% BSA at the concentrations stated, and the effects of compounds were compared to control (extracellular solution containing 0.1% BSA) parameters within the same cell. For the Ca_V_2.2 assay, CVID was incubated for 1 min before the voltage protocols were applied at 10 s intervals. Cells were maintained at a holding potential of –100 mV and Ca^2+^ currents were elicited by 120 ms depolarizing voltage steps from –100 mV to 0 mV. The current-voltage relationship of Ca_V_2.2 currents was obtained by measuring steady-state Ca^2+^ currents elicited by step depolarizations from –60 to +50 mV with 5 mV increments.

The extracellular recording solution of the Na_V_1.7 assay contained (in mM): 1 CaCl_2_, 1 MgCl_2_, 5 HEPES, 3 KCl, 140 NaCl, 0.1 CdCl_2_, 20 TEA-Cl 20; pH 7.3, adjusted with TEA-OH; osmolarity 320 mOsm. The intracellular solution contained (in mM): 140 CsF, 1 EGTA, 5 CsOH, 10 HEPES, 10 NaCl; pH 7.3, adjusted with CsOH; osmolarity 320 mOsm. The Na_V_1.7 blocker, TTX was diluted in extracellular recording solution at the concentrations stated, and the effects of compounds were compared to control (extracellular solution containing 0.1% BSA) parameters within the same cell. For Na_V_1.7 assay, TTX was incubated for 2 min at 10 s intervals. Cells were maintained at a holding potential –80 mV and Na^+^ currents elicited by 20 ms voltage steps to 0 mV from a –120 mV conditioning prepulse applied for 200 ms. The current-voltage relationship of Na_V_1.7 was obtained by measuring steady-state Na^+^ currents elicited by step depolarizations from –60 to +50 mV with 5 mV increments. Data analysis was performed using QPatch Assay Software v5.6.4 (Sophion Bioscience A/S).

### Data analysis

Data were plotted and analyzed using GraphPad Prism v7.0 (GraphPad Software Inc., San Diego, CA, USA). Sigmoidal curves for the calculation of the half-maximal excitatory concentration (EC_50_) and half-maximal inhibitory concentration (IC_50_) values were fitted to individual data points for concentration-responses by a four-parameter logistic Hill equation and Boltzmann sigmoidal equation for the voltage dependence of activation analysis. The response over baseline for FLIPR^TETRA^ was calculated by plotting ΔF/F_0_ values, where F_0_ is the baseline level of fluorescence, and ΔF is the change in fluorescence from the baseline level after the addition of agonist/antagonist. Data were represented as mean ± SEM from at least N = 3 independent experiments. Statistical significance was determined by one-way ANOVA with Dunnett’s post-test where P < 0.05 was considered significant (*).

## Results

### Evaluation of transfection efficiency and protein expression using fluorescent microscope

**Fig 3:**
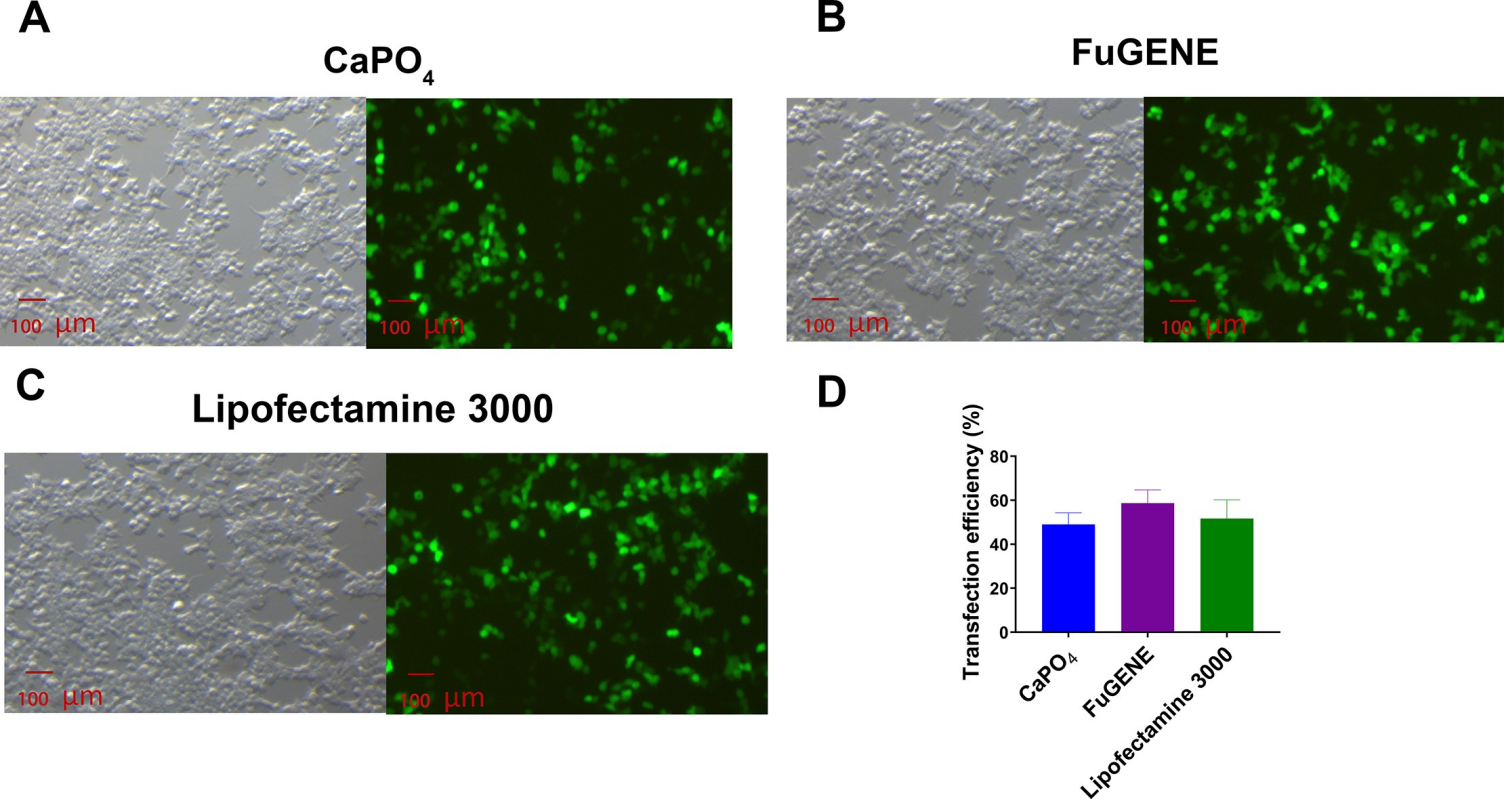
eGFP-visualized transfection. Fluorescence images of HEK 293T cells co-transfected with eGFP together with Ca_V_2.2 subunits (α_1B_, α_2_δ_1_ and β_1_) cDNA using (A) calcium phosphate, (B) FuGENE or (C) Lipofectamine 3000 was used to monitor transfection efficiency. (D) The transfection efficiency of the calcium phosphate-, FuGENE- and Lipofectamine 3000-mediated transfection was 49.0 ± 3.1%, 58.7 ± 3.5% and 51.7 ± 4.9%, respectively. Data are represented as mean ± SEM (N = 3 independent experiments conducted in triplicate).

Transient transfection and recombinant expression were verified by co-transfecting the eGFP plasmid along with the Ca_V_2.2 subunit plasmids (α_1B_, α_2_δ_1_ and β_1_) using the calcium phosphate, FuGENE and Lipofectamine 3000 methods. All transfected cells visualized under a fluorescent microscope showed strong fluorescence, indicating successful transfection was achieved using all the methods tested (Fig 3A–3C). The transfection efficiency of the calcium phosphate, FuGENE and Lipofectamine 3000-mediated transfections measured by the eGFP fluorescence intensity was 49.0 ± 3.1% (N = 3), 58.7 ± 3.5% (N = 3) and 51.7 ± 4.9% (N = 3), respectively (Fig 3D).

### Optimization of transfection

**Fig 4:**
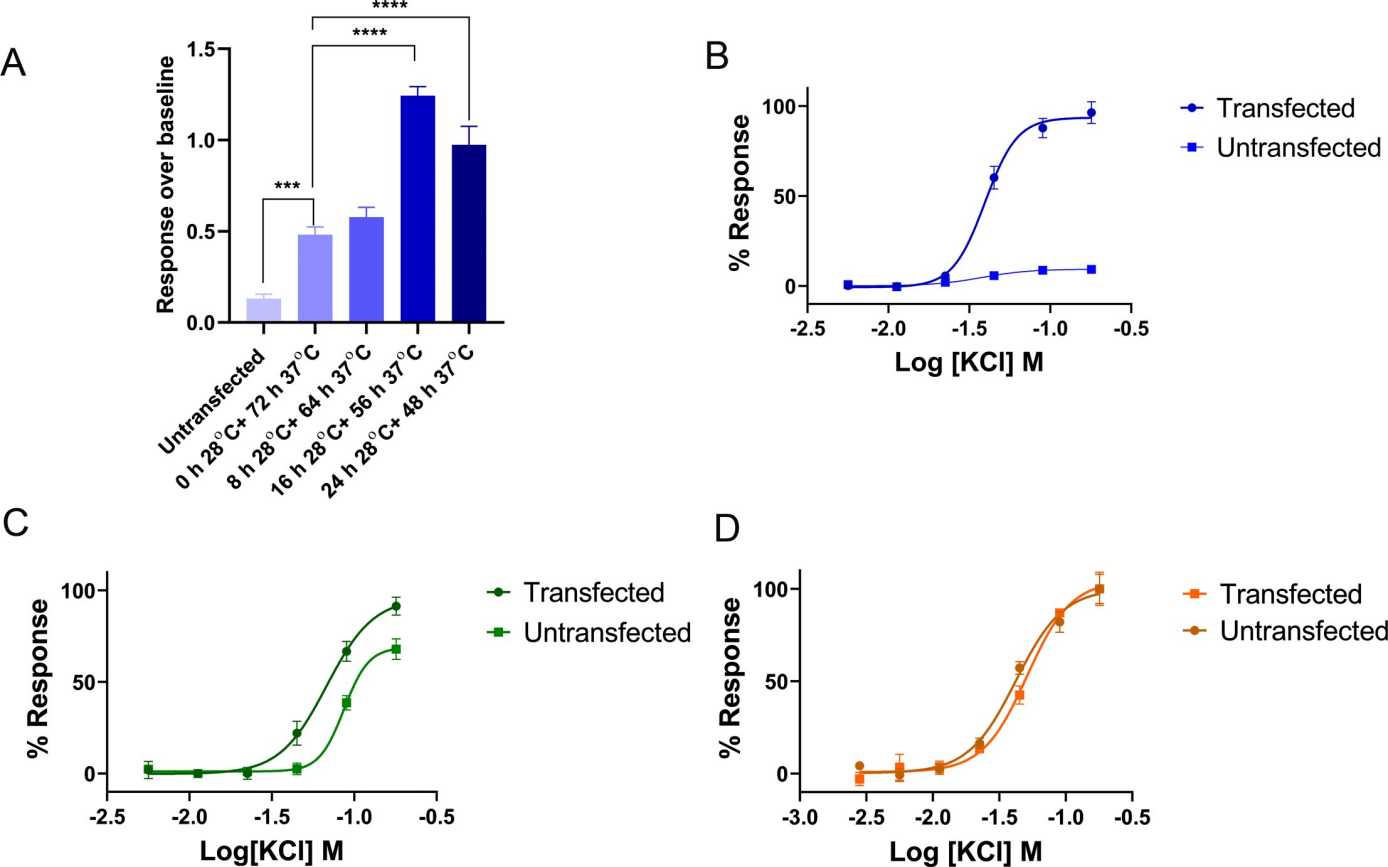
The optimization of transient transfection. (A) The comparison of 90 mM KCl-evoked fluorescence response from HEK293T cells transfected with Ca_V_2.2, incubated in four different temperature conditions. The response over baseline from untransfected cells was 0.12 ± 0.0, which was significantly lower compared to transfected cells. The response over baseline from transfected cells incubated 72h at 37°C, 8h at 28°C plus 64h at 37°C, 16h at 28°C plus 56h at 37°C, and 24h at 28°C plus 48h at 37°C were 0.5 ± 0.0, 0.6 ± 0.0, 1.2 ± 0.0, and 1.0 ± 0.1. (B) The concentration response curve of KCl-evoked Ca^2+^ response from transfected and untransfected HEK 293T cells, EC_50_ = 39.7 ± 0.3 mM for transfected cells. (C) The concentration response curve of KCl-evoked Ca^2+^ response from transfected and untransfected COS-1 cells, EC_50_ = 68.7 ± 1.2 mM for transfected cells. (D) The concentration response curve of KCl-evoked Ca^2+^ response from transfected and untransfected CHO-K1 cells, EC_50_ = 42.5 ± 2.2 mM for transfected cells. Transfected COS-1 and CHO-K1 cells gave similar response to the corresponding untransfected cells due to low expression level. Data are represented as mean ± SEM (N = 3 independent experiments conducted in triplicate), *** and **** denotes P < 0.001 and P < 0.0001, respectively.

To identify the most suitable conditions for transient transfection, initially, we transfected Ca_V_2.2 in three cell lines, HEK 293T, CHO-K1, and COS-1 using three levels of cell confluency, 40%, 60% and 80%, two incubation temperatures, 37°C and 28°C, three DNA vs transfection reagent (FuGENE) ratio, 1:2, 1:3 and 1:6, two incubation lengths, 48h and 72 h, and tested on FLIPR^TETRA^. The fluorescence response over baseline evoked from the addition of 90 mM KCl was measured. The most substantial improvement of fluorescence response was observed when we changed the incubation condition from 72h at 37°C to 16h at 37°C plus 56h at 28°C (Fig 4A). Concentration dependent KCl-evoked Ca^2+^ response was observed from transfected HEK 293T, COS-1 and CHO-K1 cells (Fig 4B–4D). Untransfected COS-1 (Fig 4C) and CHO-K1 (Fig 4D) cells elicited similar response to the corresponding transfected cells which indicates very low level of expression of Ca_V_2.2 in these two cell lines. Nonetheless, the background response from untransfected HEK 293T cells were negligible compared to transfected HEK 293T cells (Fig 4B). Based on these initial findings, we chose HEK 293T cells for the following investigations, and transfections were initiated when cells reached ~ 60% confluency using 1:3 DNA vs FuGENE and then incubated in 5% humidified CO_2_ for 16 h at 37°C followed by an additional 56 h at 28°C. The same conditions were applied to Lipofectamine 3000 and calcium phosphate transfection, with DNA vs transfection reagent ratio adjusted as per description in the method section.

### Evaluation of transiently transfected Ca_V_2.2 and Na_V_1.7 channels using fluorescence imaging in FLIPR^Tetra^

**Fig 5:**
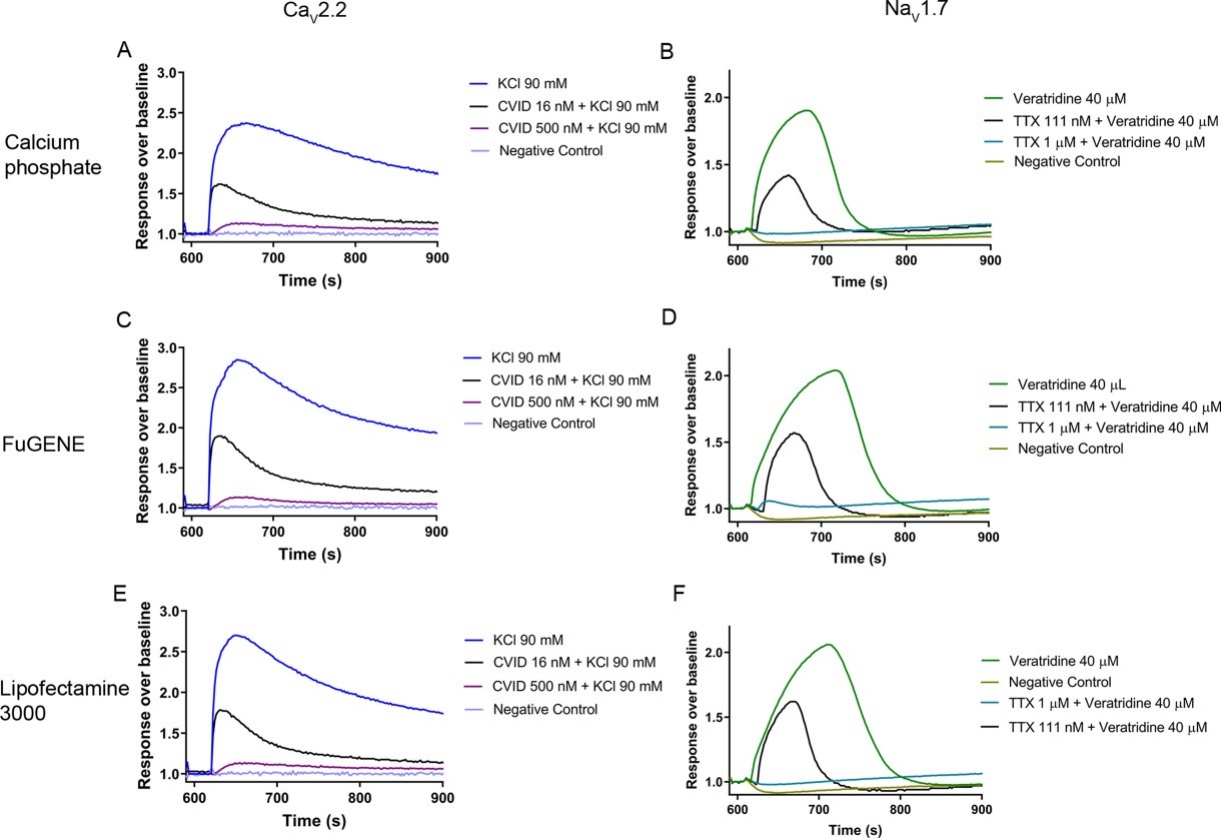
Representative Ca^2+^ fluorescence responses for Ca_V_2.2 (A, C, E) and Na_V_1.7 (B, D, F), transfected by calcium phosphate-, FuGENE-, and Lipofectamine 3000 measured in a FLIPR^TETRA^. The KCl-evoked Ca^2+^ fluorescence responses of Ca_V_2.2 over baseline for calcium phosphate-, FuGENE-, and Lipofectamine 3000 were 1.3 ± 0.0 (A), 1.7 ± 0.1 (C), and 1.5 ± 0.1 (E), respectively. The addition of 500 nM, and 16 nM CVID blocked the fluorescence response by 99.4 ± 1.8%, and 51.2 ± 3.3%, respectively, for calcium phosphate (A), 100.8 ± 0.9% and 50.1 ± 1.2%, respectively, for FuGENE (C), and 99.1 ± 1.0% and 49.5 ± 2.1%, respectively, for Lipofectamine 3000 (E). The veratridine-evoked Ca^2+^ fluorescence responses of Na_V_1.7 over baseline for calcium phosphate-, FuGENE-, and Lipofectamine 3000-mediated transfetion were 0.9 ± 0.0 (B), 1.2 ± 0.1 (D), and 1.1 ± 0.1 (E), respectively. Addition of 1 μM, and 111 nM TTX blocked the fluorescence response by 98.9 ± 1.8% and 53.8 ± 1.6%, respectively, for calcium phosphate (B), 99.8 ± 0.4% and 54.2 ± 2.0%, respectively, for FuGENE (D), and 99.4 ± 1.1% and 51.2 ± 2.3%, respectively, for Lipofectamine 3000 (F). Data are represented as mean ± SEM (N = 3 independent experiments conducted in triplicate).

**Fig 6:**
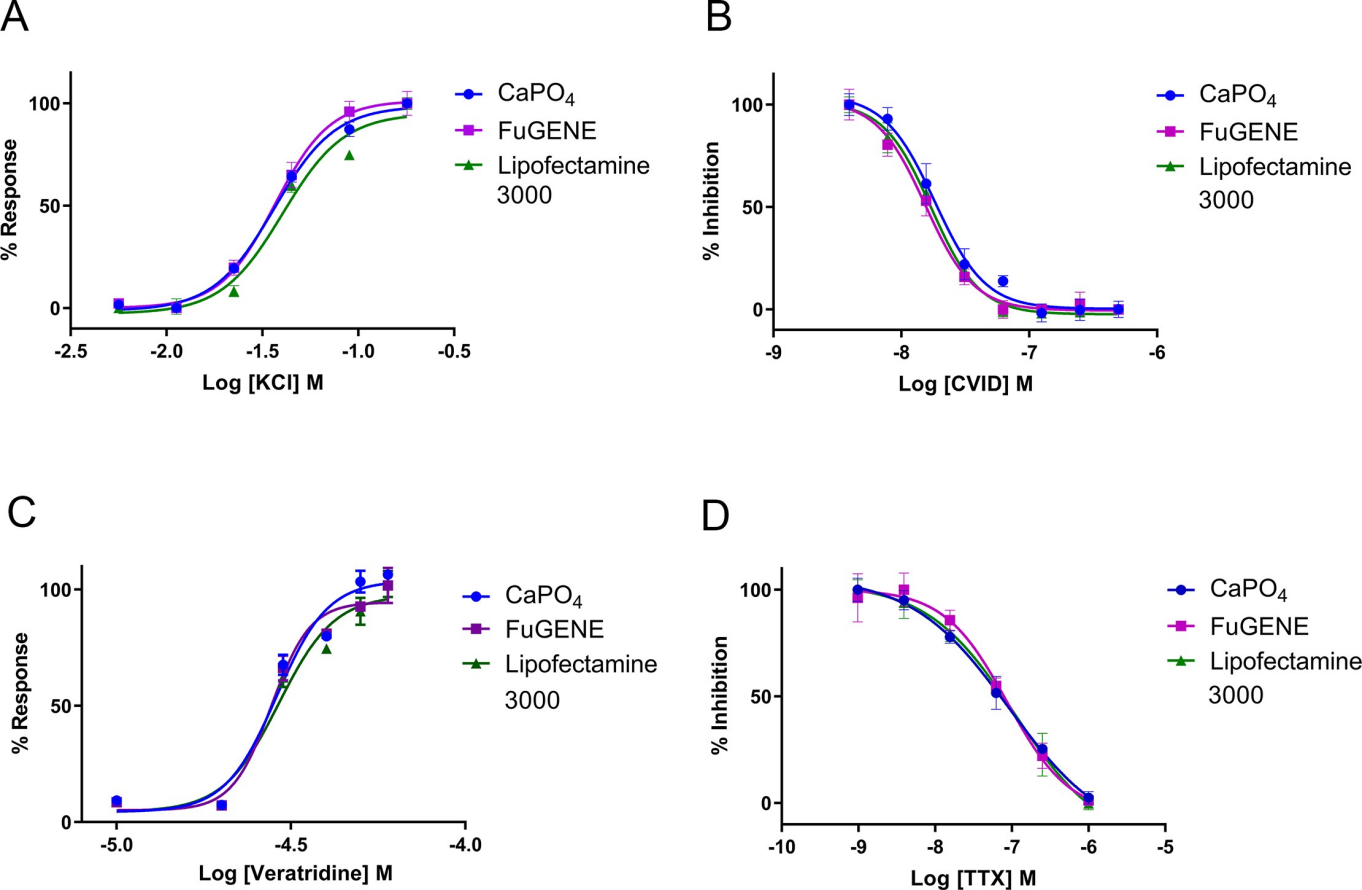
(A) Representative concentration response curves of the KCl-evoked Ca_V_2.2- responses in HEK 293T cells transfected by calcium phosphate (EC_50_ 38.1 ± 2.3 mM), FuGENE (EC_50_ 36.1 ± 0.3 mM) and Lipofectamine 3000 (EC_50_ 39.7 ± 0.3 mM). (B) Representative concentration response curves of CVID inhibition of 90 mM KCl-evoked Ca_V_2.2 responses in HEK 293T cells transfected by calcium phosphate (IC_50_ 19.5 ± 0.6 nM), FuGENE (IC_50_, 15.2 ± 1.6 nM) and Lipofectamine 3000 (IC_50_ 15.5 ± 1.1 nM). (C) Representative concentration response curves of veratridine-evoked Na_V_1.7 responses in HEK 293T cells transfected by calcium phosphate (EC_50_ 28.6 ± 0.4 μM), FuGENE (EC_50_ 28.9 ± 0.7 μM) and Lipofectamine 3000 (EC_50_ 29.9 ± 1.0 μM). (D) Representative concentration response curves of TTX inhibition of 40 μM veratridine evoked activation of Na_V_1.7 in HEK 293T cells transfected by calcium phosphate (IC_50_ 98.1 ± 3.4 nM), FuGENE (IC_50_ 95.4 ± 7.0 nM) and Lipofectamine 3000 (IC_50_ 101.7 ± 5.4 nM). Data are represented as mean ± SEM (N = 3 independent experiments conducted in triplicate).

Ca_V_2.2 and Na_V_1.7 channel activation and inhibition were evaluated using a FLIPR-based assay (Fig 5). For Ca_V_2.2 channel activation, intracellular Ca^2+^ increment was measured in response to increasing concentrations of KCl. The response over baseline for Ca_V_2.2 activation from calcium phosphate-, FuGENE-, and Lipofectamine 3000-mediated transfected cells were similar (Fig 5A, 5C and 5E) and complete inhibition of the Ca_V_2.2 response was evident from fluorescence traces recorded in the presence of 500 nM CVID. The EC_50_ of KCl-evoked activation from calcium phosphate-, FuGENE-, and Lipofectamine 3000-mediated transfected cells was 38.1 ± 2.3 mM (N = 3), 36.1 ± 0.3 mM (N = 3) and 39.7 ± 0.3 mM (N = 3), respectively (Fig 6A). The IC_50_ for CVID inhibition of these responses for calcium phosphate-, FuGENE- and Lipofectamine 3000-mediated transfected cells was 19.5 ± 0.6 nM (N = 3), 15.2 ± 1.6 nM (N = 3) and 15.5 ± 1.1 nM (N = 3), respectively (Fig 6B).

For Na_V_1.7 activation, the changes in membrane potential were measured in response to increasing concentrations of veratridine. The response over baseline for Na_V_1.7 activation from calcium phosphate-, FuGENE-, and Lipofectamine 3000-mediated transfected cells were similar (Fig 5B, 5D and 5F) and complete inhibition of responses was achieved in the presence of 1 μM TTX. The EC_50_ of veratridine-evoked activation from calcium phosphate, FuGENE and Lipofectamine 3000-mediated transfected cells was 28.6 ± 0.4 μM (N = 3), 28.9 ± 0.7 μM (N = 3) and 29.9 ± 1.0 μM (N = 3), respectively (Fig 6C). For Na_V_1.7 inhibition, increasing concentrations of TTX were added to block 40 μM veratridine-evoked responses. The IC_50_ for TTX inhibition of calcium phosphate, FuGENE and Lipofectamine 3000 transfected cells was 98.1 ± 3.4 nM (N = 3), 95.4 ± 7.0 nM (N = 3) and 101.7 ± 5.4 nM (N = 3), respectively (Fig 6D). Finally, Z’ factors were in the range of 0.50 ‒ 0.60 for the Ca_V_2.2 assays and 0.60 ‒ 0.70 for the Na_V_1.7 assays.

### Evaluation of transiently transfected CaV2.2 and NaV1.7 channels using automated whole-cell patch-clamp in QPatch 16X

**Fig 7:**
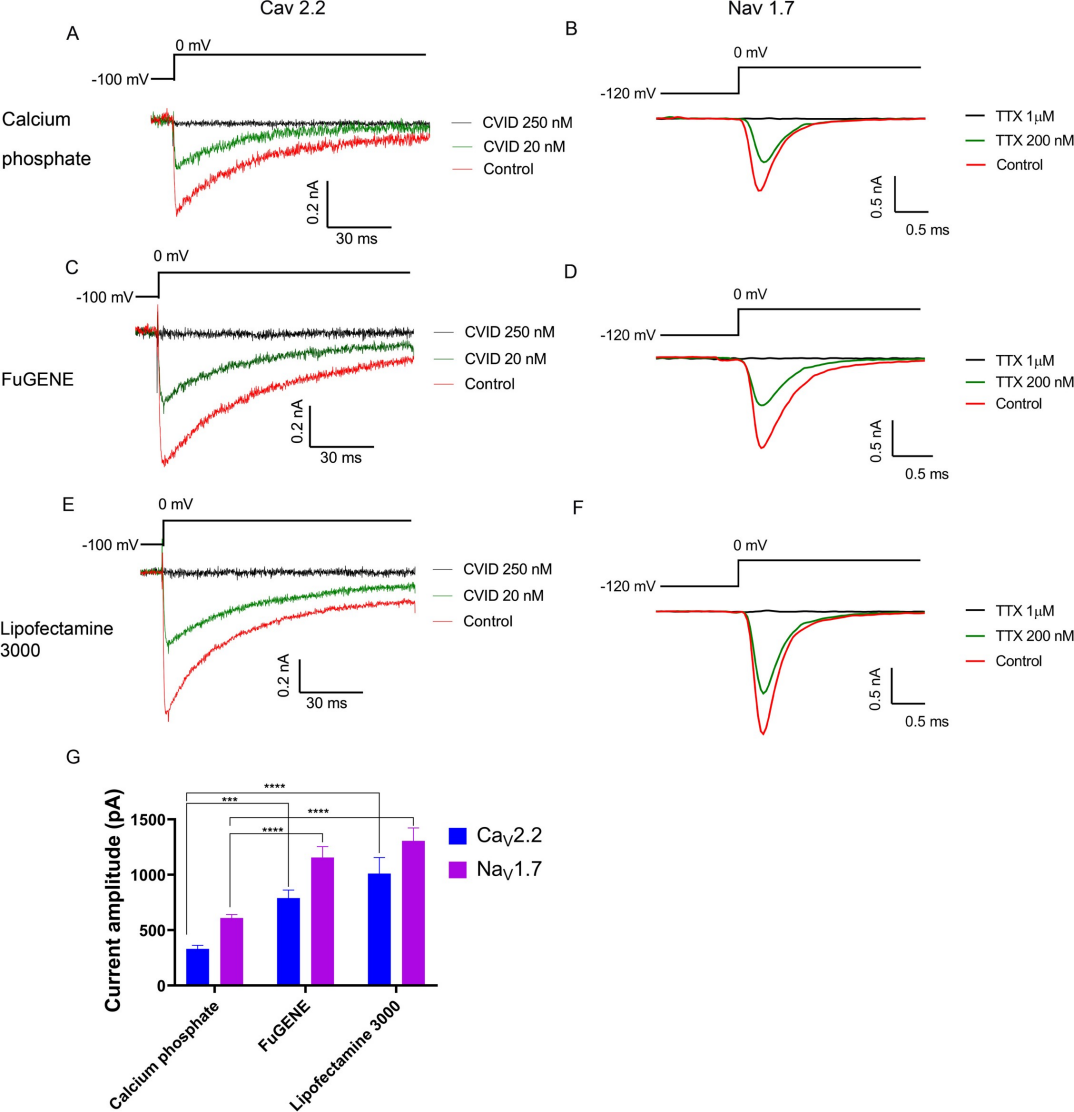
Calcium current measured with a QPatch. Representative I_Ca_ during 120 ms depolarization to V_max_ (0 mV) from a holding potential of –100 mV before and after perfusion of 20 nM and 250 nM CVID obtained from HEK 293T cells transfected by (A) calcium phosphate, (C) FuGENE or (E) Lipofectamine 3000. Representative I_Na_ during 20 ms depolarization to V_max_ (0 mV) from a holding potential of –80 mV and pre-pulse of –120 mV before and after perfusion of 200 nM and 1 μM TTX obtained from HEK 293T cells transfected by (B) calcium phosphate, (D) FuGENE or (F) Lipofectamine 3000. The current amplitude of both FuGENE-, and Lipofectamine 3000-mediated transfection methods was significantly higher compared to calcium phosphate-mediated transfection for both Ca_V_2.2 and Na_V_1.7 channels (G). Data are presented as mean ± SEM, from N = 3 independent experiments, each testing 2‒6 cells, *** and **** denotes P < 0.001 and P < 0.0001, respectively.

We additionally characterized transiently expressed Ca_V_2.2 and Na_V_1.7 using whole-cell patch-clamp in the automated electrophysiology platform QPatch 16X (Fig 7). For the Ca_V_2.2 assay, the current sizes of the calcium phosphate-, FuGENE-, and Lipofectamine 3000-mediated transfected cells were 330 ± 33 pA, 790 ± 74 pA and 1010 ± 147 pA, respectively (Fig 7G), whereas for the Na_V_1.7 assay, the current size of calcium phosphate-, FuGENE-, and Lipofectamine-mediated transfected cells produced currents of 609 ± 33 pA, 1156 ± 100 pA and 1307± 118 pA, respectively (Fig 7G). The chip, seal and whole-cell resistance values for the Ca_V_2.2 and Na_V_1.7 assays are summarized in S1 Table.

For all transient transfection methods tested, the Ca_V_2.2 currents were partially blocked by addition of 20 nM CVID and fully blocked by addition of 250 nM CVID (Fig 7A, 7C and 7E), whereas the Na_V_1.7 currents were partially blocked by addition of 200 nM TTX and fully blocked by addition of 1 μM (Fig 7B, 7D and 7F).

**Fig 8:**
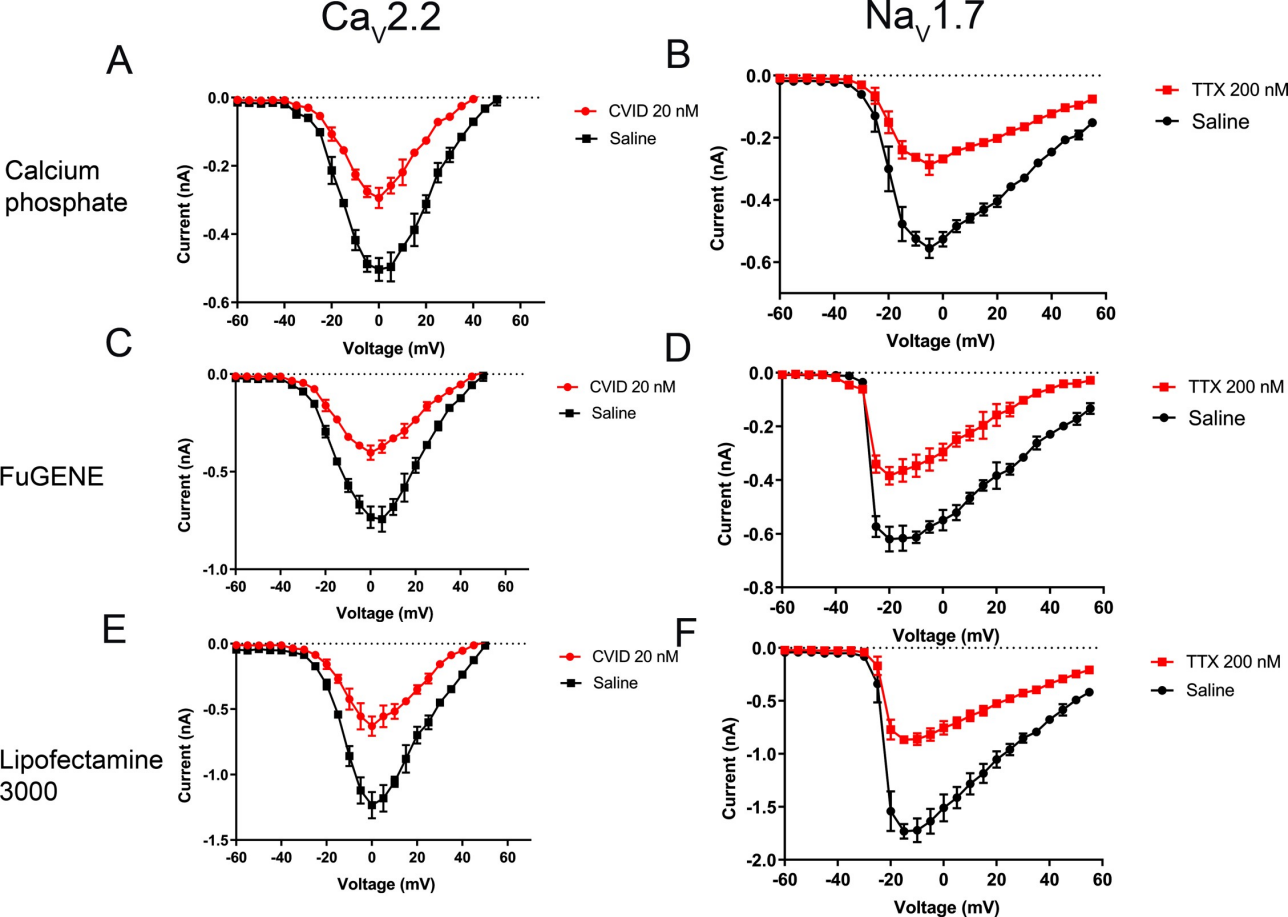
Current-voltage relationship of Ca_V_2.2 in the presence and absence of 20 nM CVID from HEK 293T cells transfected by (A) calcium phosphate, (C) FuGENE and (E) Lipofectamine 3000. Ca_V_2.2-mediated currents were evoked by depolarization steps from a holding potential of –100 mV followed by –60 mV to +50 mV in 5-mV increment. Current-voltage relationship of Na_V_1.7 in the presence and absence of 200 nM TTX obtained from HEK 293T cells transfected by (B) calcium phosphate, (D) FuGENE and (F) Lipofectamine 3000. Na_V_1.7-mediated currents were evoked by step depolarization from a holding potential of –80 mV followed by –60 mV to +55 mV in 5-mV increment. Data are presented as mean ± SEM, with N  =  4–9 cells per data point.

The current-voltage relationships of Ca_V_2.2 and Na_V_1.7 were also determined (Fig 8). The current-voltage relationship of Ca_V_2.2 showed a partial block of the peak current in the presence of 20 nM CVID and no shift of V_max_ was observed in these transfected cells compared to the known physiological properties of this channel (Fig 8A, 8C and 8E). Similarly, the current-voltage relationship of Na_V_1.7 showed a partial block of the current in the presence of 200 nM TTX, and no shift of V_max_ was observed in these transfected cells (Fig 8B, 8D and 8F).

We tested 24 cells using calcium phosphate-, FuGENE-, and Lipofectamine 3000-mediated transfection and success rates were measured by determining the percentage of cells that successfully completed the experiment and produced reasonable Ca_V_2.2 or Na_V_1.7 currents (Table 1). The FuGENE-mediated transfection had the best success rate as it was significantly higher compared with the Lipofectamine 3000-mediated transfection method (Table 1).

**Table 1:** Success rates of different transfection methods in the QPatch 16X showing mean ± SEM from N = 3 independent experiments, each testing 8 cells (24 cells per total).

Ion channel	Transfection method	Cells in whole-cell configuration	Cells with current	Success rate (%)
**Ca_V_2.2**	Calcium phosphate	15	8	29.2 ± 4.2
	FuGENE	17	11	45.8 ± 8.3*
	Lipofectamine 3000	9	5	20.8 ± 4.2
**Na_V_1.7**	Calcium phosphate	14	9	37.5 ± 13
	FuGENE	15	12	50.0 ± 7.2*
	Lipofectamine 3000	10	6	25.0 ± 7.2

* P < 0.05, compared to Lipofectamine

## Discussion

Recent developments in HT cellular assay methods have produced innovative reagents and instruments that allow the rapid exploration of ion channel pharmacology and the discovery of novel pharmacological tools and analgesics targeting ion channels [31, 32]. These include indicator dyes for fluorescence imaging HT assays using the FLIPR^Tetra^ (Molecular Devices) [12, 13, 33], and automated electrophysiology multi-well systems that allow accurate voltage-protocols and high-quality GΩ seals in HT assays, such as the next generation QPatch II (Sophion Bioscience A/S) and the PatchXpress (Molecular Devices).

The increasing number of ion channels and receptors for investigation require transient transfection methods for screening programs as well as structure-function studies. This work presents the first systematic study of chemical transfection methods for transient expression of the pain-related ion channels Ca_V_2.2 and Na_V_1.7 and compared their efficiencies using both fluorescence and electrophysiological HT cellular assay platforms. We observed that whereas all transfection methods tested showed similar efficiency in FLIPR assays, the automated electrophysiology in QPatch showed the FuGENE-mediated transfection with best success rates for both Ca_V_2.2 and Na_V_1.7 channels. These findings indicate that variations in transfection efficiencies and/or cytotoxicity associated with different transfection methods are not reflected in fluorescent assays using multiple cells. At high channel activity dyes can be saturated leading to non-linear readout of channel activity. Most probably, the difference falls outside the dynamic range of the assay. On the other hand, whole-cell patch-clamp assays using a single cell per measurement are largely dependent on the transfection efficiency as well as of the cytotoxicity elicited by the transfection procedures, which often impacts membrane stability and whole-cell configuration.

Chemical transient transfection remains the most popular method for functional studies of ion channels due to the cost-effectiveness and ready availability, including heteromultimeric ion channels such as Ca_V_2.2, which require the co-expression of three subunits (Ca_V_α, Ca_V_β and Ca_V_α_2_δ) to obtain physiological function [34]. The calcium phosphate precipitation method has been commonly used for traditional patch clamp studies of transiently expressed VGICs [35–37], whereas FuGENE-, and Lipofectamine 3000-mediated transfection methods have been reported to use for transient expression of VGICs as well as for other receptors like G protein-coupled receptors [38–41]. However, the application of chemical transient transfection methods for HT platforms remains limited.

The FLIPR^Tetra^ platform allows high-throughput screening of ion channels and receptors by measuring fluorescence emission from thousands of cells per well in either 96-wells or 384-wells format [12]. Our FLIPR^Tetra^ assay results using KCl and veratridine for Ca_V_2.2 and Na_V_1.7 activation, and CVID and TTX for Ca_V_2.2 and Na_V_1.7 inhibition, respectively, did not show differences among the three transfection methods tested. Therefore, low to average transient channel expression amongst thousands of cells was sufficient to achieve a reasonable signal to noise and produce useful assays.

The IC_50_ values for CVID and TTX inhibition was ranging from 15‒20 nM, and 98‒100 nM respectively, for three different transfection methods when tested on FLIPR. However, in previous studies, the reported IC_50_ value was 50‒70 pM for CVID inhibition of Ca_V_2.2 response in radioligand binding assays [42], and 35‒40 nM for TTX inhibition of Na_V_1.7 responses in manual patch clamp assays [43]. This difference in potency might be attributed to the effect of spare receptors after the saturation of Ca^2+^ sensitive dye in FLIPR.

Automated patch clamp platforms such as the QPatch have also been successfully applied in HT ion channel drug discovery [44]. These platforms harness a range of commercially available stable cell lines expressing VGICs and receptors, that are validated in these HT assays [44–46]. On the other hand, only one report has studied chemically-transfected cells in HT automated patch clamp using FuGENE [40]. In these automated systems, the unguided selection of cells leads to a number of whole-cells displaying no currents and/or poor membrane integrity, hence broad and efficient and transfection across cells is essential to achieve reasonable success rates. We observed that Lipofectamine 3000-mediated transfection provided the lowest success rates in QPatch for both channels, as demonstrated by the lowest number of cells reaching whole-cell configuration with detectable ion currents. Thus, Lipofectamine 3000-mediated transfection appears sub-optimal for studies of VGICs using automated electrophysiological HT platforms, potentially due to its lipidic nature, which affect membrane stability and whole-cell seal formation. However, when transfection and whole-cell formation were achieved, Lipofectamine 3000 produced the larger current for both Ca_V_2.2. and Na_V_1.7 channels, suggesting the delivery of higher quantities of foreign DNA into the cells compared to calcium phosphate and FuGENE. The ability to produce larger currents could be useful in studies of ion channels and receptors that show limited responses in HT assay systems.

The FuGENE-mediated transfection performed the best in QPatch assays, showing 46 and 50% success rates for both Ca_V_2.2 and Na_V_1.7 channels, respectively. The FuGENE reagent is a cationic polymer that forms complexes with nucleic acid named polyplexes or micelles in aqueous solutions, which are often less toxic for the cell membrane [47]. Such ability to penetrate the cell membrane to efficiently deliver foreign DNA and protein expression, and maintain the integrity and stability of the membrane is key for the successful use of this reagent in whole-cell patch-clamp HT assays. Although the calcium phosphate transfection method also showed superior whole-cell formation rates compared to the Lipofectamine 3000 method, its ability to deliver foreign DNA into the cells was evidently less as observed by the smaller ion current sizes compared to the other methods tested.

In conclusion, the widely studied pain-related VGICs Ca_V_2.2 and Na_V_1.7 have been subjected to a systematic evaluation of appropriate transfection methods for applications in HT cellular assays platforms. Whereas the calcium phosphate precipitation method remains preferable for FLIPR assays due to its cost-effectiveness, the FuGENE-mediated transfection is the optimum choice for automated whole-cell electrophysiology in QPatch. These results provide a guide for future applications of transient ion channel expression applied to HT platforms and therefore accelerate studies of structure-function of ion channels and the discovery of novel drugs targeting VGICs as alternatives to opioid drugs to treat chronic pain.

## Supporting information

S1 Table Comparison of peak currents and resistance values from cells transfected with different transfection methods and tested in QPatch 16X assays. (DOCX)
