# Supplementary material for: Transfection methods for high-throughput cellular assays of voltage-gated calcium and sodium channels involved in pain
Source: PLoS One. 2021 Mar 5;16(3):e0243645. doi: 10.1371/journal.pone.0243645 (PMC7935312; doi:10.1371/journal.pone.0243645)
Supplement: S1 Table — (DOCX) [file pone.0243645.s001.docx]

**S1 Table.**

| Transfection  method | Ca_V_2.2 | | Na_V_1.7 | |
| --- | --- | --- | --- | --- |
|  | **Peak current amplitude (pA)**  **(Mean ± SEM)** | **Chip, Seal, Whole-cell resistance (MΩ)**  **(Mean ± SEM)** | **Peak current amplitude (pA)**  **(Mean ± SEM)** | **Chip, Seal, Whole-cell resistance (MΩ)**  **(Mean ± SEM)** |
| Calcium phosphate | 330 ± 33.29 (*n* = 8) | 1.73 ± 0.02, 951.38 ± 66.39 and 835.88 ± 69.54 (*n* = 8) | 609.44 ± 32.96 (*n* = 9) | 2.22 ± 0.02, 897.63 ± 78.96 and 742.375 ± 65.12 (*n* = 9) |
| FuGENE | 789.09 ± 73.96 (*n* = 11) | 1.79 ± 0.16, 1316.73 ± 130.37 and 1041.91 ± 58.66 (*n* = 11) | 1155.83 ± 99.63 (*n* = 12) | 1.98 ± 0.04, 1048.33 ± 54.07 and 975.25 ± 48.9 (*n* = 12) |
| Lipofectamine 3000 | 1010 ± 146.86 (*n* = 5) | 1.76 ± 0.01, 1166.60 ± 103.1 and 1067.5 ± 57.84 (*n* = 5) | 1306.66 ± 117.89 (*n* = 6) | 2.04 ± 0.06, 961.8 ± 86.31 and 847.8 ± 81.63 (*n* = 6) |
